# Prevalence of Posttraumatic Stress Disorder in Prisoners

**DOI:** 10.1093/epirev/mxx015

**Published:** 2018-03-27

**Authors:** Gergõ Baranyi, Megan Cassidy, Seena Fazel, Stefan Priebe, Adrian P Mundt

**Affiliations:** 1 Center for Research on Environment Society and Health, School of GeoSciences, The University of Edinburgh, Edinburgh, United Kingdom; 2 Unit for Social and Community Psychiatry, WHO Collaborating Center for Mental Health Services Development, Queen Mary University of London, London, United Kingdom; 3 Department of Psychiatry, University of Oxford, Oxford, United Kingdom; 4 Medical Faculty, Universidad Diego Portales, Santiago, Chile; 5 Medical School, Universidad San Sebastián, Puerto Montt, Chile

**Keywords:** mental health, meta-analysis, posttraumatic, prisoners, review, stress disorders

## Abstract

People involved with criminal justice frequently are exposed to violence and traumatic experiences. This may lead to posttraumatic stress disorder (PTSD); however, no review, to our knowledge, has synthetized findings in this setting. We conducted a systematic review and meta-analysis to estimate prevalence rates of PTSD in prison populations. Original studies in which prevalence rates of PTSD in unselected samples of incarcerated people were reported were systematically searched between 1980 and June 2017. Data were pooled using random-effects meta-analysis, and sources of heterogeneity for prespecified characteristics were assessed by meta-regression. We identified 56 samples comprising 21,099 imprisoned men and women from 20 countries. Point prevalence of PTSD ranged from 0.1% to 27% for male, and from 12% to 38% for female prisoner populations. The random-effects pooled point prevalence was 6.2% (95% confidence interval: 3.9, 9.0) in male prisoners and 21.1% (95% confidence interval: 16.9, 25.6) in female prisoners. The heterogeneity between the included studies was very high. Higher prevalence was reported in samples of female prisoners, smaller studies (*n* < 100), and for investigations based in high-income countries. Existing evidence shows high levels of PTSD among imprisoned people, especially women. Psychosocial interventions to prevent violence, especially against children and women, and to mitigate its consequences in marginalized communities must be improved. Trauma-informed approaches for correctional programs and scalable PTSD treatments in prisons require further consideration.

## INTRODUCTION

The size of the prison population has grown worldwide over the last 2 decades. In 2015, there were more than 10.3 million people imprisoned (1). Women constitute 6.8% of the total prison population, and their proportion is rising in most countries (1). Prevalence rates of severe mental disorders are higher in imprisoned people than in the general population: Approximately 4% of those in jails and prisons are estimated to have psychotic illnesses, and greater than 10% of male and 14% of female prisoners are reported to have major depression (2). Prevalence rates of substance use disorders (3) and personality disorders (4) also have been found to be much higher in the prison population than in the general population. However, the prevalence of posttraumatic stress disorder (PTSD) in prison populations is not reliably known, despite several individual reports in which authors have stated PTSD is a major health problem in prisoners because of high rates of exposure to physical, sexual, and emotional violence in imprisoned people over their lifespan (5–7). Untreated PTSD reduces day-to-day functioning and adherence to treatment (8), and increases the risks of self-harm and suicide (9). In one study, 90% of the imprisoned people with PTSD were reported to have unmet needs for psychiatric care, including pharmacotherapy and psychological treatment, which is the highest rate among all mental disorders (10). PTSD treatments during imprisonment may have the potential to reduce barriers to complete correctional interventions, improve adherence to medical treatments, and improve the longer-term institutional and community rehabilitation.

To our knowledge, 1 systematic review (11) has been published on the prevalence of PTSD in sentenced prison populations; it included only 4 studies published until 2004, and the study authors suggested the prevalence of PTSD in sentenced prisoners is likely to be higher than in the general population and that women were disproportionately affected. The low number of studies precluded synthesizing the data. Since 2004, several prevalence studies of mental health problems in people involved with the criminal justice system have been published from a range of countries (2). The aim of the present work was to review systematically the literature on PTSD in prison populations worldwide, estimate the prevalence, and assess the heterogeneity between studies.

## METHODS

This systematic review was conducted following the Meta-analysis of Observational Studies in Epidemiology guidelines (12) and the Preferred Reporting Items for Systematic Reviews and Meta-Analyses (13). The research protocol was registered on the Prospero database (CRD42015020899).

### Search strategies

**Figure 1.**
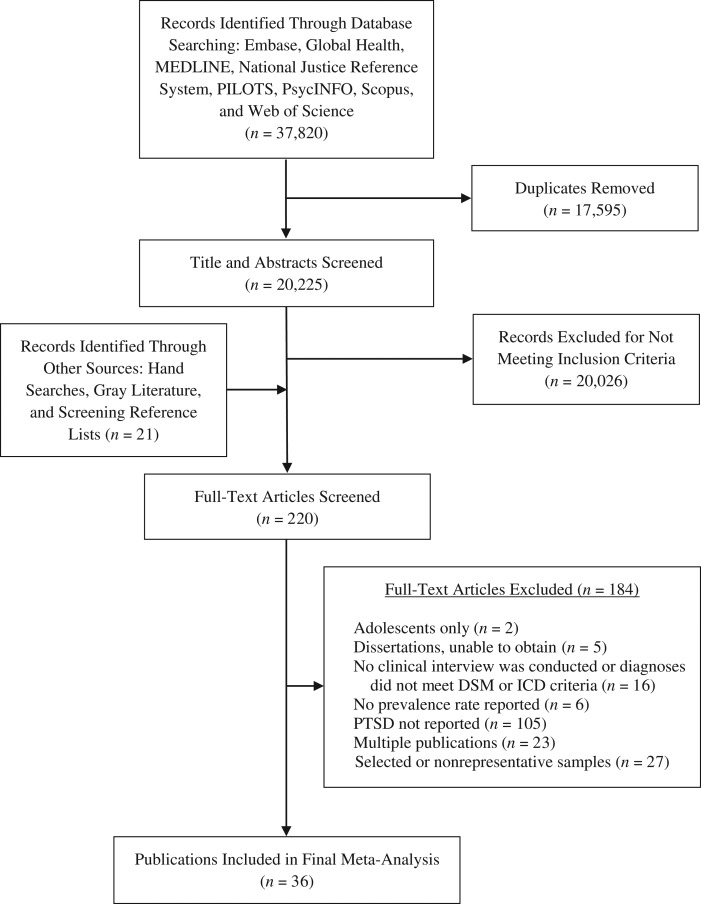
Flow diagram showing the different stages of finding relevant studies on the prevalence of posttraumatic stress disorder in prison populations between 1980 and 2017.

We conducted a systematic search of the literature covering the time from 1980, when PTSD as a diagnostic category was introduced in the *Diagnostic and Statistical Manual of Mental Disorders, Third Edition* (14), until June 2017. The search included the following: 1) online databases (i.e., Embase, Global Health, MEDLINE, and PsycINFO via OvidSP; PILOTS via ProQuest; National Justice Reference System; Scopus; Web of Science); 2) key journals (i.e., *Criminal Behavior and Mental Health*, *International Journal of Law and Psychiatry*, *Journal of Traumatic Stress*); 3) reference lists of identified papers and relevant systematic reviews (2, 11); and 4) unpublished literature (OpenGrey) and correspondence with authors (Figure 1).

For the online database searches, we used the following combined strategy of free-text strings and subject headings, indicated as [SH] in medical subject headings searches, related to 1) PTSD (Anxiety disorders [SH] OR Mental* OR Posttraumatic stress OR Post-traumatic stress OR Psych* OR PTSD OR Stress Disorders, Post-Traumatic [SH] OR Stress Disorders, Traumatic, Acute [SH] OR Stress disorder OR Stress reaction*); 2) prison settings (Correctional OR Custod* OR Detain* OR Detention OR Forensic Psychiatry [SH] OR Gaol* OR Imprison* OR Incarcerat* OR Inmate* OR Jail* OR Offend* OR Penal OR Prison* OR Prisons [SH] OR Prisoners [SH] OR Probat* OR Remand OR Sentenced); and 3) prevalence studies (Epidemiolog* OR Epidemiology [SH] OR Population* OR Prevalence [SH] OR Prevalence). Language restrictions were not applied. Non-English articles were translated.

### Inclusion and exclusion criteria

We identified studies in which prevalence rates of PTSD in the general prison population were reported. The following inclusion criteria were applied: 1) data were collected from unselected general prison populations; 2) PTSD diagnoses were established with validated instruments as part of a clinical or research interview; and 3) PTSD diagnoses met the criteria of international classifications (i.e., of the *Diagnostic and Statistical Manual of Mental Disorders* or *International Classification of Diseases*). Studies meeting the following criteria were excluded: 1) selection of 1 particular age group, such as adolescents, or a particular offender type (15); 2) reporting diagnoses based on self-report questionnaires and/or medical records (16); 3) 2-stage sampling (i.e., screening and diagnostic interview), because this approach might lead to different prevalence estimates owing to the characteristics of the screening instruments (17); 4) not reporting the criterion of a traumatic event, key symptoms (i.e., re-experiencing, avoidance, hyperarousal) or a minimum symptom persistence (18); 5) no separate reports for female and male participants (19), unless the proportion of 1 sex represented less than 10% of the entire sample, in which case the study was considered representative for 1 sex (≥90%) (20, 21); and 6) when more than 1 publication reported data from the same sample, only the most comprehensive publication was retained.

One author (G.B.) screened abstracts and full texts. Another (M.C.) independently screened 20% of the studies. Disagreements between these 2 reviewers were resolved by consensus with a third reviewer (A.P.M.).

### Data extraction

Two reviewers (G.B., M.C.) independently extracted information from the included studies. The following data were extracted: sex, mean age, legal status (i.e., remand, sentenced, or mixed), year of publication, year and country of data collection, sampling method, diagnostic instruments, diagnostic classification (according to *Diagnostic and Statistical Manual of Mental Disorders* or *International Classification of Diseases*), sample size, refusal rate, and number of imprisoned individuals diagnosed with PTSD. The following period(s) covered by the reported prevalence estimates were extracted: point (from point up to 6 months), 1-year, and lifetime prevalence rates. When relevant data were missing or further clarification was needed, the authors were contacted.

When the refusal rate was not available, the nonresponse rate was used. It was prespecified that samples with refusal rates of less than 15% were considered as low and samples with refusal rates of 15% or greater were consider as high in refusal rates (2). Because prevalence estimates may relate to the sample size, we subdivided the included samples into small (*n* ≤ 200) and large samples (*n* > 200). Samples with fewer than 100 participants were aggregated for meta-analyses by summarizing the numerators and denominators (2). When the year of data collection was not reported, we imputed a year on the basis of the mean difference between the year of publication and data collection (3 years). We included remand prisoners (i.e., jail inmates, pretrial, and/or awaiting sentencing) and sentenced prisoners. We classified the sample as remand or sentenced when greater than 90% of the study sample belonged to the corresponding category; otherwise, the sample was classified as mixed.

There is preliminary evidence for geographical differences in the prevalence estimates of mental disorders in prison populations (2). To account for possible differences, we classified the country of data collection in the included studies as a high-income country (HIC) or low- or middle-income country (LMIC), using the World Bank classification based on the gross national income per capita at the time of data collection. Because most of the studies were conducted in the United States, which has the largest prison population in the world (1), we also classified the country of data collection as United States versus any other country (non-US).

### Quality assessment

The included studies were subject to quality appraisal by 2 reviewers (G.B., M.C.) who used a modified checklist, based on the Newcastle-Ottawa Scale (22), from a meta-analysis on the prevalence of depression (23). It assesses representativeness, sample size, participation rate, interviewer and quality of descriptive statistics (Web Appendix 1, available at https://academic.oup.com/aje). The total ratings range was from 0 to 6 points. Samples were considered of low quality if they scored from 0 to 2 points; medium quality, from 3 to 4 points; and high quality, from 5 to 6 points (ratings of all included samples are available in Web Table 1).

### Statistical analysis

When prevalence estimates were separately reported for male and female, remand, sentenced and mixed, and cross-sectional and admission samples, they were acknowledged in the statistical analyses as different samples. As a consequence, the number of samples may be higher than the number of studies. Because prevalence estimates may differ according to the time covered in the interview, separate meta-analyses were conducted for point, 1-year, and lifetime prevalence rates of PTSD. To acknowledge the high heterogeneity between the samples, we used random-effects models to balance the weighting of studies for data syntheses (24). We used Wilson’s method (25) to calculate 95% confidence intervals for prevalence estimates. The approach produces asymmetric confidence intervals in studies with low prevalence rates. Freeman-Tukey double arcsine transformation was used to stabilize the variance before pooling (25). This procedure makes the normal distribution assumption more applicable to significance testing when the number is small and/or the proportion is close to the margins 0 and 1. Heterogeneity among studies was estimated based on Cochran Q and reported using *I*^2^ (and 95% confidence interval of the *I*^*2*^). *I*^2^ > 75% is considered indicative of high heterogeneity (26). We conducted sensitivity analyses including only high-quality studies and tested whether this reduced the overall heterogeneity.

Random-effects meta-regressions were conducted to assess the effects of sample characteristics on the prevalence rates of PTSD. When significant associations (*P* < 0.05) with PTSD prevalence rates were indicated in univariate analyses results, we retained variables for multivariate models. We calculated adjusted *R*^*2*^ as the proportion of variance between the samples that was explained by the variables. For significant study characteristics, prevalence rates were estimated in subgroup analyses. Meta-analyses were conducted with aggregated smaller samples (*n* < 100); meta-regressions were based on individual samples.

To explore publication bias, we first inspected funnel plots of the prevalence estimates against standard errors, and then conducted Egger regression tests to assess the symmetry of the funnel plots; these were performed separately for male and female samples. Differences of the study quality and small study bias were explored as possible reasons for asymmetric funnel plots (27). Statistical analyses were conducted with Stata, version 13 (StataCorp LP, College Station, Texas).

## RESULTS

Thirty-six studies comprising 56 samples with a total of 21,099 imprisoned people met the inclusion criteria (main characteristics of the included studies are listed in Table 1; additional information is available in Web Table 2). Male prisoners constituted 76.4% (*n* = 16,111 participants) of the pooled samples; 23.6% (*n* = 4,988 participants) were female prisoners. The weighted mean age was 30.6 years. Studies were conducted in 20 countries, of which 14 were classified as HICs in the year of the data collection: Australia (28, 29), Austria (30), Canada (31–34), Chile (35), France (36), Germany (37–40), Iceland (41), Ireland (42), Netherlands (43), New Zealand (44), Spain (45, 46), Switzerland (47), United Kingdom (48, 49), and United States (50–56); and 7 were LMICs: Brazil (57), Chile (58), China (59), India (21), Iran (60), South Africa (20), and Turkey (61). The World Bank classification of Chile changed from LMIC to HIC over time.

**Table 1.** Description of Included Samples Reporting the Prevalence of Posttraumatic Stress Disorder in Prison Populations, 1980–2017

First Author, Year (Reference No.)	Country	Income Group	Sex	Sample Size	Type of Study	Sampling Method	Diagnostic Instrument	Diagnostic Criteria	Legal Status
Andreoli, 2014 (57)^a^	Brazil	LMIC	Male	676	C	Stratified random	CIDI	ICD-10	Sentenced
Andreoli, 2014 (57)^a^	Brazil	LMIC	Female	617	C	Stratified random	CIDI	ICD-10	Sentenced
Andreoli, 2014 (57)^a^	Brazil	LMIC	Male	516	C	Stratified random	CIDI	ICD-10	Remand
Assadi, 2006 (60)	Iran	LMIC	Male	351	C	Stratified random	SCID	DSM-IV	Sentenced
Beaudette, 2016 (31)^b^	Canada	HIC	Male	1,110	A	Population	SCID	DSM-IV	Sentenced
Bebbington, 2017 (48)^a^	UK	HIC	Male	197	C	Random	PDS	DSM-IV	Mixed
Bebbington, 2017 (48)^a^	UK	HIC	Female	171	C	Random	PDS	DSM-IV	Mixed
Boşgelmez, 2010 (61)^b^	Turkey	LMIC	Male	30	C	Stratified random	SCID	DSM-IV	Mixed
Boşgelmez, 2010 (61)^b^	Turkey	LMIC	Female	30	C	Stratified random	SCID	DSM-IV	Mixed
Brink, 2001 (32)	Canada	HIC	Male	202	A	Random	SCID	DSM-IV	Sentenced
Brooke, 1996 (49)	UK	HIC	Male	750	C	Stratified random	Clinical interview	ICD-10	Remand
Bulten, 2009 (43)	The Netherlands	HIC	Male	191	A	Random	MINI	DSM-III-R	Mixed
Butler, 2003 (28)	Australia	HIC	Male	756	A	Convenience	CIDI	ICD-10	Mixed
Butler, 2003 (28)	Australia	HIC	Male	458	C	Stratified random	CIDI	ICD-10	Sentenced
Butler, 2003 (28)	Australia	HIC	Female	165	A	Convenience	CIDI	ICD-10	Mixed
Butler, 2003 (28)	Australia	HIC	Female	108	C	Stratified random	CIDI	ICD-10	Sentenced
Derkzen, 2016 (34)^a^	Canada	HIC	Female	154	C	Population	SCID	DSM-IV	Sentenced
Duburcq, 2004 (36)^b^	France	HIC	Male	799	C	Stratified random	MINI	DSM-IV	Mixed
Duburcq, 2004 (36)^b^	France, MQ	HIC	Male	100	C	Stratified random	MINI	DSM-IV	Mixed
Duburcq, 2004 (36)^b^	France	HIC	Female	99	C	Stratified random	MINI	DSM-IV	Mixed
Dudeck, 2009 (37)^b^	Germany	HIC	Male	102	C	Population	SCID	DSM-IV	Sentenced
Einarsson, 2009 (41)^a^	Iceland	HIC	Male	90	A	Population	MINI	DSM-IV	Sentenced
Gunter, 2008 (50)	US	HIC	Male	264	A	Random	MINI	DSM-IV	Sentenced
Gunter, 2008 (50)	US	HIC	Female	56	A	Random	MINI	DSM-IV	Sentenced
Guthrie, 1998 (51)	US	HIC	Male	100	C	Random	CAPS	DSM-IV	Sentenced
Hodgins, 1990 (33)	Canada	HIC	Male	495	C	Random	DIS	DSM-III	Sentenced
Huang, 2006 (59)	China	LMIC	Female	471	C	Random	CAPS	DSM-IV	Sentenced
Lynch, 2014 (52)^a^	US	HIC	Female	233	C	Random	CIDI	DSM-IV	Sentenced
Lynch, 2014 (52)^a^	US	HIC	Female	249	C	Random	CIDI	DSM-IV	Remand
Math, 2011 (21)	India	LMIC	Male	1,197	A and C	Population	MINI	DSM-IV and ICD-10	Sentenced
Math, 2011 (21)	India	LMIC	Male	3,827	A and C	Population	MINI	DSM-IV and ICD-10	Remand
Mir, 2015 (40)	Germany	HIC	Female	150	A	Population	MINI	DSM-IV	Mixed
Missoni, 2003 (38)^a^	Germany	HIC	Male	107	A	Population	DIA-X	ICD-10	Remand
Mohan, 1997 (42)	Ireland	HIC	Female	45	A	Random	SCAN	DSM-IV	Mixed
Mundt, 2013 (58)^a^	Chile	LMIC	Male	855	C	Stratified random	CIDI	DSM-IV	Mixed
Mundt, 2013 (58)^a^	Chile	LMIC	Female	153	C	Stratified random	CIDI	DSM-IV	Mixed
Mundt, 2016 (35)	Chile	HIC	Male	229	A	Systematic	MINI	DSM-IV	Mixed
Mundt, 2016 (35)	Chile	HIC	Female	198	A	Population	MINI	DSM-IV	Mixed
Naidoo, 2012 (20)	South Africa	LMIC	Male	120	C	Stratified random	MINI and ICD-10	DSM-IV and ICD-10	Sentenced
Naidoo, 2012 (20)	South Africa	LMIC	Male	73	C	Stratified random	MINI	DSM-IV and ICD-10	Remand
Powell, 1997 (53)	US	HIC	Male	118	C	Stratified random	DIS	DSM-III-R	Sentenced
Powell, 1997 (53)	US	HIC	Male	95	C	Stratified random	DIS	DSM-III-R	Remand
Simpson, 1999 (44)	New Zealand	HIC	Male	645	C	Stratified random	CIDI	DSM-IV	Sentenced
Simpson, 1999 (44)	New Zealand	HIC	Male	441	C	Population	CIDI	DSM-IV	Remand
Simpson, 1999 (44)	New Zealand	HIC	Female	162	C	Population	CIDI	DSM-IV	Sentenced
Stompe, 2010 (30)	Austria	HIC	Male	100	A	Population	SCAN	ICD-10	Sentenced
Stompe, 2010 (30)	Austria	HIC	Male	100	A	Population	SCAN	ICD-10	Remand
Teplin, 1996 (54)	US	HIC	Female	1,272	A	Stratified random	DIS	DSM-III-R	Remand
Trestman, 2007 (55)	US	HIC	Male	306	A	Systematic	CAPS	DSM-IV	Remand
Trestman, 2007 (55)	US	HIC	Female	199	A	Systematic	CAPS	DSM-IV	Remand
Tye, 2006 (29)	Australia	HIC	Female	103	C	Population	CIDI	ICD-10	Mixed
Urbaniok, 2007 (47)	Switzerland	HIC	Male	25	C	Population	PDS	DSM-IV	Remand
Vicens, 2011 (46)	Spain	HIC	Male	707	C	Stratified random	SCID	DSM-IV	Sentenced
von Schönfeld, 2006 (39)	Germany	HIC	Female	63	C	Population	SCID	DSM-IV	Sentenced
Zabala-Baños, 2016 (45)^a^	Spain	HIC	Male	184	C	Stratified random	SCID	DSM-IV	Sentenced
Zlotnick, 1997 (56)	US	HIC	Female	85	C	Random	SCID	DSM-IV	Sentenced

Abbreviations: A, admission; C, cross-sectional; CAPS, Clinician-Administered Posttraumatic Stress Disorder Scale for Diagnostic and Statistical Manual of Mental Disorders; CIDI, Composite International Diagnostic Interview; DIA-X, Diagnostic Expert System for Mental Disorders; DIS, Diagnostic Interview Schedule; DSM, *Diagnostic and Statistical Manual of Mental Disorders*; DSM-III-R, *Diagnostic and Statistical Manual of Mental Disorders, Third Edition, Revised*; DSM-IV, *Diagnostic and Statistical Manual of Mental Disorders, Fourth Edition*; HIC, high-income country; ICD-10, *International Statistical Classification of Diseases, Tenth Revision*; LMIC, low- or middle-income country; MINI, Mini-International Neuropsychiatric Interview; MQ, Martinique; PDS, Posttraumatic Stress Diagnostic Scale; SCAN, Schedules for Clinical Assessment in Neuropsychiatry; SCID, Structured Clinical Interview for Diagnostic and Statistical Manual of Mental Disorders; UK, United Kingdom; US, United States.

^a^ Authors provided additional data.

^b^ Authors provided additional information about their studies.

### Point prevalence of PTSD

**Figure 2.**
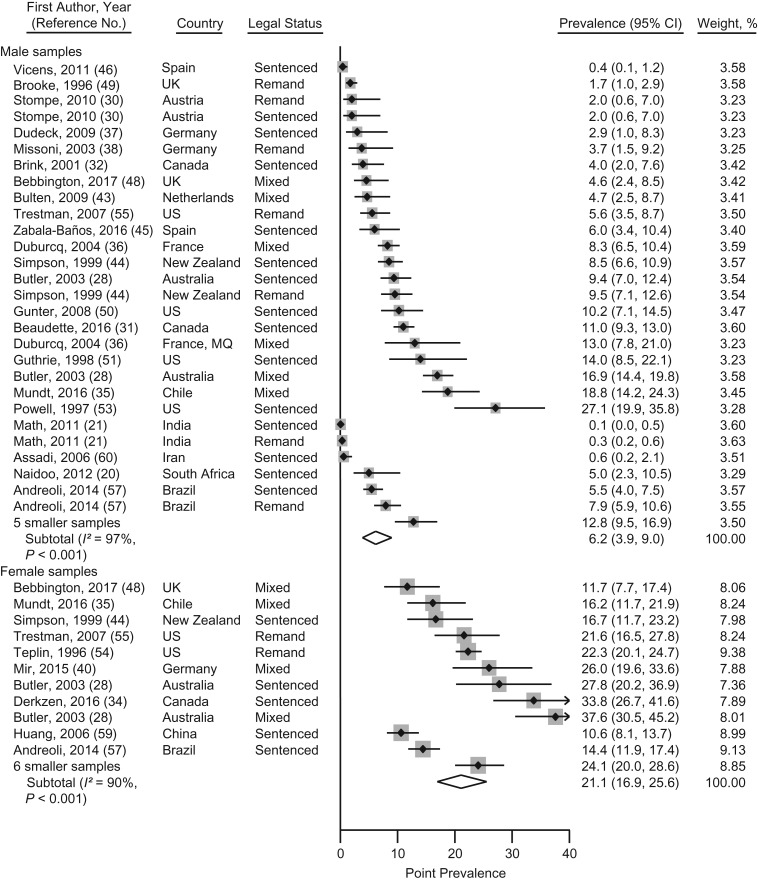
Prevalence meta-analysis of point prevalence of posttraumatic stress disorder in male and female prisoners from studies published between 1980 and 2017. Samples are sorted by sex and high- versus low- or middle-income countries, as well as by ascending prevalence rates within the subgroups. Sample weights from random-effects meta-analyses may not sum to 100, because of rounding errors. Smaller samples (*n* < 100) were aggregated for male (20, 41, 47, 53, 61) and female subgroups (36, 39, 42, 50, 56, 61). CI, confidence interval; MQ, Martinique; UK, United Kingdom; US, United States.

Point prevalence of PTSD were reported in 50 samples from 20 countries including 19,011 participants (20, 21, 28, 30–32, 34–51, 53–57, 59–61). Aggregating small samples (*n* < 100), the point prevalence estimates of PTSD ranged from 0.1% to 27% for male prisoners, and from 12% to 38% for female prisoners. The pooled point prevalence was 6.2% (95% confidence interval (CI): 3.9, 9.0) in male prisoners and 21.1% (95% CI: 16.9, 25.6) in female prisoners (Figure 2). Heterogeneity was high for male (*I*^2^ = 97%, 95% CI: 97, 98) and female (*I*^2^ = 90%, 95% CI: 85, 94) samples. Excluding low- and medium-quality studies in sensitivity analysis, the heterogeneity was lower in male samples (92%, 95% CI: 90, 94), which reduced the pooled point prevalence to 5.9% (95% CI: 4.0, 8.2). The heterogeneity in the female samples did not materially change (Web Table 3).

Univariate meta-regression analyses showed significant sex differences in the point prevalence of PTSD: Female prisoners had higher prevalence rates than did male prisoners (β = 0.138, standard error of β (SE(β)) = 0.024, *P* < 0.001). We also found significantly higher rates in samples from HICs as compared with LMICs (β = −0.072, SE(β) = 0.030, *P* = 0.02), and specifically in samples from the United States (β = −0.093, SE(β) = 0.036, *P* = 0.01). There was a significant association of the sample size with the point prevalence of PTSD: Large studies (*n* > 200) had lower rates than small studies (β = −0.077, SE(β) = 0.027, *P* = 0.006). All other variables (e.g., legal status, diagnostic classification) were not significantly associated with the point prevalence of PTSD (Table 2).

**Table 2.** Meta-Regression Analyses Assessing Prespecified Sample Characteristics as Possible Sources of Heterogeneity for the Prevalence of Posttraumatic Stress Disorder in Prisoner Populations

Variable^a^	Point Prevalence of PTSD			1-Year Prevalence of PTSD			Lifetime Prevalence of PTSD		
	β	SE (β)	*P* Value^b^	β	SE	*P* Value^b^	β	SE	*P* Value^b^
*Univariate Meta-Regression Analyses*									
Age of prisoners (continuous)	−0.003	0.004	0.49	−0.005	0.018	0.79	−0.004	0.012	0.76
Year of data collection (continuous)	−0.002	0.002	0.40	−0.010	0.010	0.34	0.001	0.005	0.77
Sex: male vs. female	0.138	0.024	<0.001	0.156	0.067	0.04	0.227	0.051	<0.001
Country group 1: HIC vs. LMIC	−0.072	0.030	0.02	−0.200	0.049	0.002	−0.009	0.091	0.92
Country group 2: US vs. non-US	−0.093	0.036	0.01	–^c^			−0.198	0.064	0.006
Sampling: cross-sectional vs. admission	0.002	0.029	0.94	–^c^			−0.072	0.084	0.40
Penal status: sentenced vs. remand	−0.013	0.034	0.71	–^c^			0.067	0.079	0.40
Diagnostic classification: DSM vs. ICD	−0.014	0.032	0.66	0.071	0.087	0.44	0.059	0.101	0.56
Refusal rate: low (≤15%) vs. high (>15%)	0.020	0.027	0.47	0.191	0.052	0.001	0.074	0.074	0.33
Sample size: small (*n* ≤ 200) vs. large (*n* > 200)	−0.077	0.027	0.006	–^c^			−0.102	0.075	0.19
*Multivariate Meta-Regression Analyses Retaining Significant Variables*									
Sex: male vs. female	0.119	0.023	<0.001				0.181	0.052	0.003
Country group 1: HIC vs. LMIC	−0.057	0.021	0.008						
Country group 2: US vs. non-US	−0.037	0.028	0.18				−0.115	0.057	0.06^d^
Sample size: small (*n* ≤ 200) vs. large (*n* > 200)	−0.019	0.023	0.40						

Abbreviations: DSM, *Diagnostic and Statistical Manual of Mental Disorders*; HIC, high-income country; ICD, *International Statistical Classification of Diseases*; LMIC, low- or middle-income country; PTSD, posttraumatic stress disorder; SE, standard error; US, United States.

^a^ The reference category is given first.

^b^ Statistical significance set at *P* < 0.05.

^c^ Insufficient number of samples in at least 1 of the comparison groups.

^d^*P* for trend is statistically significant at < 0.1.

Significant predictors of the point prevalence of PTSD in univariate analyses were retained in a multivariate model. Sex (β = 0.119, SE(β) = 0.023, *P* < 0.001) and income group of the country (β = −0.057, SE(β) = 0.021, *P* = 0.008) remained significantly associated with the point prevalence of PTSD: Higher rates were reported for female prisoners and for samples from HICs (Table 2). The results of the model accounted for 89% of the between-sample variance. Following the results of the multivariate meta-regression, we estimated the sex-specific point prevalence of PTSD for the samples from HICs. The pooled point prevalence of PTSD was 7.5% (95% CI: 5.2, 10.2) in male samples and 23.3% (94% CI: 19.3, 27.7) in female samples from HICs. The heterogeneity was high in male (*I*^2^ = 94%, 95% CI: 92, 96) and female samples (*I*^2^ = 83%, 95% CI: 71, 90) from HICs. According to the results of the Egger test for asymmetry of funnel plots, a significant bias may exist in the male samples reporting point prevalence estimates of PTSD (*P* = 0.009), whereas there was no strong evidence for bias in the female samples (*P* = 0.49) (Web Figure 1).

### One-year prevalence of PTSD

The 1-year prevalence of PTSD was reported in 12 samples from 4 countries including 4,889 participants (28, 29, 52, 57, 58). The 1-year prevalence estimates ranged from 1% to 22% for male prisoners and from 3% to 44% for female prisoners. Pooled 1-year prevalence estimates of PTSD were 9.9% (95% CI: 3.0, 20.2) for male prisoners and 26.1% (95% CI: 15.9, 37.8) for female prisoners. Heterogeneity was very high among male (*I*^2^ = 99%, 95% CI: 98, 99) and female samples (*I*^2^ = 96%, 95% CI: 93, 97). The heterogeneity did not significantly change when excluding low- and medium-quality samples from sensitivity analyses (Web Table 3).

By univariate meta-regression analyses assessing the heterogeneity, we found a significantly higher prevalence among female prisoners (*P* = 0.04) and among HICs as compared with LMICs (*P* = 0.002). High refusal rates also were associated with a higher 1-year prevalence (*P* = 0.001) (Table 2). Multivariate analyses were not conducted owing to the small number of samples. According to the results of the Egger test, funnel plots were not asymmetric in male (*P* = 0.56) and female samples (*P* = 0.22) reporting 1-year prevalence rates of PTSD (Web Figure 2).

### Lifetime prevalence of PTSD

**Figure 3.**
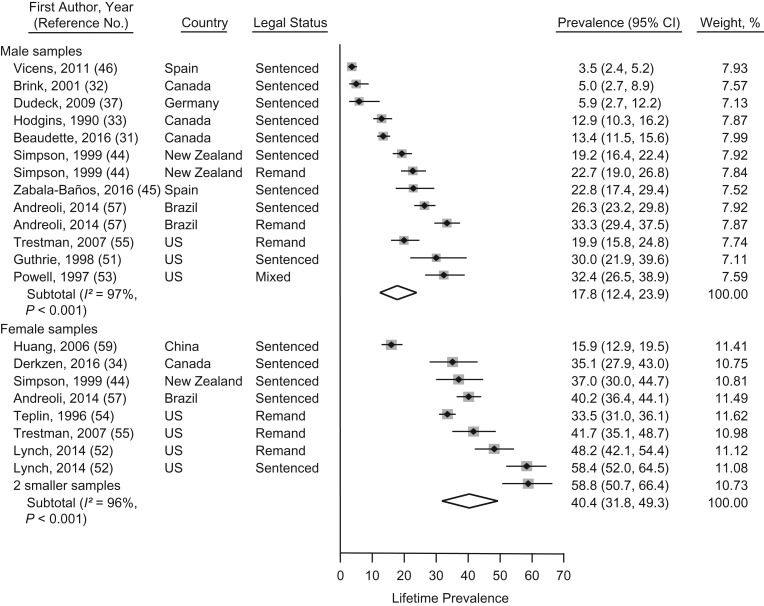
Prevalence meta-analysis of lifetime prevalence rates of posttraumatic stress disorder in male and female prisoners from studies published between 1980 and 2017. Samples are sorted by sex and non-US versus United States, as well as by ascending prevalence rates within the subgroups. Sample weights from random-effects meta-analysis may not sum to 100, because of rounding errors. Smaller female samples (*n* < 100) were aggregated (39, 56). CI, confidence interval; UK, United Kingdom; US, United States.

The lifetime prevalence of PTSD in prison populations was reported for 23 samples from 7 countries including 9,202 individuals (31–34, 37, 39, 44–46, 51–57, 59). Aggregating smaller samples (*n* < 100), lifetime prevalence estimates ranged from 4% to 32% in male and from 16% to 58% in female prisoners. Pooled lifetime prevalence estimates of PTSD were 17.8% (95% CI: 12.4, 23.9) in male prisoners and 40.4% (95% CI: 31.8, 49.3) in female prisoners (Figure 3). Heterogeneity was very high between male (*I*^2^ = 97%, 95% CI: 96, 98) and female samples (*I*^2^ = 96%, 95% CI: 94, 97). Excluding low- and medium quality samples in sensitivity analyses did not significantly change the heterogeneity in male and female samples (Web Table 3).

There were significant sex differences between male and female prisoners for the lifetime prevalence estimates in univariate meta-regression analyses (β = 0.227, SE(β) = 0.051, *P* < 0.001). In studies from the United States, significantly higher lifetime PTSD prevalence estimates were reported as compared with studies from other countries (β = −0.198, SE(β) = 0.064, *P* = 0.006) (Table 2).

According to results of multivariate meta-regression analyses including sex and country effects in a single model, lifetime prevalence of PTSD was significantly higher in female than male prisoners (β = 0.181, SE(β) = 0.052, *P* = 0.003). There was a trend for higher lifetime prevalence rates of PTSD in samples from the United States compared with samples from other countries (β = −0.115, SE(β) = 0.057, *P* = 0.06) (Table 2). Using the model, we could explain 62% of the between-sample variance. The sex-specific lifetime prevalence of PTSD was estimated for the samples from the United States: Pooled lifetime prevalence estimates of PTSD were 27.0% (95% CI: 18.7, 36.2) in male and 49.5% (95% CI: 37.3, 61.8) in female samples from the United States. The heterogeneity remained high in male (*I*^2^ = 82%, 95% CI: 46, 94) and female (*I*^2^ = 96%, 95% CI: 92, 97) samples. According to results of the Egger test, funnel plots were not asymmetric in male (*P* = 0.78) and female (*P* = 0.16) samples in which the lifetime prevalence of PTSD was reported (Web Figure 3).

## DISCUSSION

This systematic review of the prevalence of PTSD in prison populations is based on 56 samples from 20 countries worldwide. Point, 1-year, and lifetime prevalence rates indicate high levels of PTSD in this population. Imprisoned women have prevalence rates of PTSD that are approximately 3-fold than those in men. Prisoners in HICs and, in particular, in the United States, had higher a PTSD prevalence than did imprisoned people in other countries. When data were pooled, the point prevalence of PTSD was 6% in male prison populations and 21% in female prison populations, the 1-year prevalence rates of PTSD were 10% in male and 26% in female, and the lifetime prevalence estimates of PTSD were 18% in male and 40% in female prison populations.

To our knowledge, this is the first review of the prevalence of PTSD in prisoners that includes a sufficient number of samples to perform data synthesis, meta-analyses, and quantitative assessment of sources for heterogeneity. The overall evidence is based on more than 21,000 assessments of individuals in prison. In this study, we provide pooled prevalence estimates for point, 1-year, and lifetime prevalence of PTSD in prison populations. Some limitations need to be highlighted. First, comorbid conditions of PTSD with other mental health and substance use disorders were typically not reported in the studies and not taken into account in this review, even though they may be important for clinical decisions and service development. Second, the high level of heterogeneity between samples could not be explained solely by the examined study characteristics and warrants cautious interpretation of the pooled prevalence estimates. Although our review did not reveal differences between individuals who were sentenced versus those on remand, or between cross-sectional samples and studies conducted at intake in the correctional system, it is possible that some subgroups, such as those returned on breaches or in solitary confinement, have higher rates of PTSD. Additional study characteristics, such as sampling techniques and the different diagnostic interviews used to assess PTSD, were not tested in our models, because of the small number of samples in each of the different groups, although they may explain part of the heterogeneity. Hence, an alternative approach would be to report prevalence ranges, which are provided.

Imprisoned people are mostly young, poor, and have substance use problems; many have histories of child abuse and are highly marginalized (7). Therefore, compared with the general population, several risk factors for PTSD are increased and high rates of PTSD may not be surprising. Nevertheless, a comparison with the general population may be helpful to contextualize the prevalence data reported here. According to studies conducted in Western countries, the point prevalence of PTSD has been estimated to be 1.2% in men and 2.7% in women (62), and the lifetime prevalence of PTSD has been estimated to be 5.0% in men and 10.4% in women in the general population (63). Compared with the general population, our results suggest an approximately 5-fold higher point prevalence of PTSD in male prisoners and an 8-fold higher point prevalence of PTSD in female prisoners. Thus, based on these findings, PTSD appears to be a common mental disorder in prison populations (7), and absolute numbers will be large. Of the 10.3 million prisoners worldwide (1), approximately 750,000 are likely to have a clinical diagnosis of PTSD. In the United States (1), this equates to more than 300,000 prisoners with PTSD, based on a current custodial population of 2.2 million. Given that most prisoners have short-term sentences, the numbers passing through prisons will be higher in any given 1-year period.

In contrast to previous reviews of mental disorders in prisoners (2, 3, 11), the present meta-analysis provides evidence for a significant sex difference in prevalence rates. PTSD in female prisoners is approximately 3 times more frequent than in male prisoners. This finding is consistent with research in the general population that indicates women are more likely to develop symptoms of PTSD after traumatic events than men and to have higher rates of PTSD (14). According to a literature review on sex differences of PTSD in community settings (64), there are several possible explanations of elevated rates in women, such as the type of trauma, age at trauma exposure, gender roles in society, and stress coping mechanisms. Female prisoners have particularly high rates of exposure to sexual violence before imprisonment and of exposure to violence during childhood (65). After experiencing a traumatic event, it is reported that women more often use maladaptive coping strategies to manage stress and trauma-related symptoms. More frequent and heavier use of substances, and passive and avoidance-focused coping styles are more common in women than in men (7, 64). In the context of increasing female prison populations in some countries, the higher prevalence of PTSD in female prisoners points toward a need to specifically consider this group in research and service development.

In addition, we have reported higher point prevalence and 1-year prevalence of PTSD in studies from HICs and higher lifetime prevalence of PTSD in studies from the United States. It is unclear whether prisoners from the United States and other HICs are more often exposed to traumatic events before or during imprisonment than prisoners from other countries or whether they are more likely to develop PTSD after traumatic experiences (66).

Imprisoned individuals with PTSD are more likely to have comorbid mental disorders, particularly substance use (7, 40, 56, 67), affective, and anxiety disorders (52, 67). The potential causal links between PTSD and these other disorders remain largely unclear. PTSD may be associated with a risk for repeat victimization, within and outside of the prison system (68). Moreover, exposure to violence and PTSD has been associated with violent behavior during imprisonment and elevated risks of reoffending afterward (69).

Several implications arise from our review. First, efforts to prevent child abuse and sex-related violence in marginalized populations should be improved (70). Stronger, early psychosocial interventions to mitigate traumatic experiences associated with violence and emotional abuse are needed in the community. Second, trauma-informed approaches should be considered for all correctional programs during incarceration and in the community. Rates of spontaneous long-term remission from PTSD are modest (71); therefore, methods of identifying prisoners with PTSD and providing effective treatments on a large scale would be desirable. Prisons are, in various respects, a challenging context for providing health care interventions and, for many imprisoned people, the duration of stay is not known at intake; sentences can be brief. Incarceration can also be a chance to reach people with mental disorders who otherwise might not access treatment. However, the evidence for psychological treatments in this setting is limited (72) and more trials are required to test to what extent established treatments for PTSD in community settings are effective in the prison context. In addition, trials that address how these treatments can be embedded in more complex interventions for the many treatment needs of prisoners are required. Alternatively, new and specific approaches should be developed, which may have to be group based to respond to the large unmet needs in prison populations.

## Supplementary Material

Supplementary Data

## Abbreviations

CI: confidence interval
HIC: high-income country
LMIC: low- or middle-income country
non-US: any other country other than the United States
PTSD: posttraumatic stress disorder
SE(β): standard error of β
SH: subject heading. CI, confidence interval
HIC: high-income country
LMIC: low- or middle-income country
non-US: any other country other than the United States
PTSD: posttraumatic stress disorder
SE(β): standard error of β
SH: subject heading
