# Supplementary material for: Prevalence of Posttraumatic Stress Disorder in Prisoners
Source: Epidemiol Rev. 2018 Mar 27;40(1):134–45. doi: 10.1093/epirev/mxx015 (PMC5982805; doi:10.1093/epirev/mxx015)
Supplement: Supplementary Data [file mxx015mundtwebmaterialfinal.pdf]

Web Material

Web Appendix 1. Quality Appraisal Scale

Web Table 1. Quality Scoring of Included Samples, 1980-2017

Web Table 2. Extracted Information of Included Samples, 1980-2017

Web Table 3. Sensitivity Analyses

Web Figure 1. Funnel Plots of Point Prevalence Estimates Against Standard Errors (for male and female samples), 1980-2017

Web Figure 2. Funnel Plots of One-year Prevalence Estimates Against Standard Errors (for male and female samples), 1980-2017

Web Figure 3. Funnel Plots of Lifetime Prevalence Estimates Against Standard Errors (for male and female samples), 1980-2017

Web Appendix 1. Quality Appraisal Scale*

(1) Representativeness of the sample:
1 point: Stratified random, random, population or systematic sampling.
0 point: Convenience sampling.

(2) Sample size:
1 point: Sample size equal to or greater than 100 participants.
0 point: Sample size less than 100 participants.

(3) Participation:
a. Reporting of participation rate or non-response analysis
1 point: Yes
0 point: No

b. Satisfactory rate of participation (if refusal rate not reported, non-response rate used)
1 point: Participation rate was equal or higher than 80%
0 point: Participation rate was lower than 80%, or not reported

(4) Validity of PTSD diagnosis:
1 point: Psychiatrist(s)/psychologist(s) made the PTSD diagnoses
0 point: Trained interviewer(s) made the PTSD diagnoses

(5) Quality of descriptive statistics:
1 point: Reporting of the descriptive statistics to describe the sample included age AND at least one other socio-demographic or criminal characteristic.
0 point: Descriptive statistics were not reported or were incomplete.

Scoring:
1-2 Low
3-4 Medium
5-6 High

*based on a modified version of the Newcastle-Ottawa Scale (22, 23)

Web Table 1. Quality Scoring of the Included Samples, 1980-2017

First Author, Year (Reference No.)	Represen-tativeness	Sample size	Reporting of participation rate	Satisfactory rate of participation	Validity of diagnostic	Quality of statistics	Total score	Quality group
Andreoli, 2014 (57) M, sentenced	1	1	1	1	0	1	5	High
Andreoli, 2014 (57) F, sentenced	1	1	1	1	0	1	5	High
Andreoli, 2014 (57) M remand	1	1	1	1	0	1	5	High
Assadi, 2006 (60)	1	1	1	1	1	1	6	High
Beaudette, 2016 (31)	1	1	1	0	0	1	4	Medium
Bebbington, 2017 (48) M	1	1	1	0	1	1	5	High
Bebbington, 2017 (48) F	1	1	1	0	1	1	5	High
Boşgelmez, 2010 (61) M	1	0	1	1	1	1	5	High
Boşgelmez, 2010 (61) F	1	0	1	1	1	1	5	High
Brink, 2001 (32)	1	1	1	1	1	1	6	High
Brooke, 1996 (49)	1	1	1	1	1	0	5	High
Bulten, 2009 (43)	1	1	1	1	1	1	6	High
Butler, 2003 (28) M, mixed	0	1	1	0	0	1	3	Medium
Butler, 2003 (28) M, sentenced	1	1	1	1	0	1	5	High
Butler, 2003 (28) F, mixed	0	1	1	0	0	1	3	Medium
Butler, 2003 (28) F, sentenced	1	1	1	1	0	1	5	High

First Author, Year (Reference No.)	Represen-tativeness	Sample size	Reporting of participation rate	Satisfactory rate of participation	Validity of diagnostic	Quality of statistics	Total score	Quality group
Derkzen, 2016 (34)	1	1	0	0	1	1	4	Medium
Duburcq, 2004 (36) M	1	1	1	0	1	1	5	High
Duburcq, 2004 (36) M, MQ	1	1	1	0	1	1	5	High
Duburcq, 2004 (36) F	1	0	1	0	1	1	4	Medium
Dudeck, 2009 (37)	1	1	1	1	1	1	6	High
Einarsson, 2009 (41)	1	0	1	1	1	1	5	High
Gunter, 2008 (50) M	1	1	1	1	0	1	5	High
Gunter, 2008 (50) F	1	0	1	1	0	1	4	Medium
Guthrie, 1998 (51)	1	1	1	0	1	1	5	High
Hodgins, 1990 (33)	1	1	1	0	0	1	4	Medium
Huang, 2006 (59)	1	1	1	1	1	1	6	High
Lynch, 2014 (52) sentenced	1	1	1	0	0	1	4	Medium
Lynch, 2014 (52) remand	1	1	1	0	0	1	4	Medium
Math, 2011 (21) sentenced	1	1	0	0	0	1	3	Medium
Math, 2011 (21) remand	1	1	0	0	0	1	3	Medium
Mir, 2015 (40)	1	1	1	0	1	1	5	High
Missoni, 2003 (38)	1	1	1	1	1	1	6	High
Mohan, 1997 (42)	1	0	1	1	1	1	5	High

First Author, Year (Reference No.)	Represen-tativeness	Sample size	Reporting of participation rate	Satisfactory rate of participation	Validity of diagnostic	Quality of statistics	Total score	Quality group
Mundt, 2013 (58) M	1	1	1	1	0	1	5	High
Mundt, 2013 (58) F	1	1	1	1	0	1	5	High
Mundt, 2016 (35) M	1	1	1	1	1	1	6	High
Mundt, 2016 (35) F	1	1	1	1	1	1	6	High
Naidoo, 2012 (20) sentenced	1	1	1	0	1	1	5	High
Naidoo, 2012 (20) remand	1	0	1	0	1	1	4	Medium
Powell, 1997 (53) sentenced	1	1	1	0	0	1	4	Medium
Powell, 1997 (53) remand	1	0	1	0	0	1	3	Medium
Simpson, 1999 (44) M, sentenced	1	1	1	0	0	1	4	Medium
Simpson, 1999 (44) M, remand	1	1	1	1	0	1	5	High
Simpson, 1999 (44) F, sentenced	1	1	1	1	0	1	5	High
Stompe, 2010 (30) sentenced	1	1	0	0	0	0	2	Low
Stompe, 2010 (30) remand	1	1	0	0	0	0	2	Low
Teplin, 1996 (54)	1	1	1	1	0	1	5	High
Trestman, 2007 (55) M	1	1	1	1	0	1	5	High
Trestman, 2007 (55) F	1	1	1	1	0	1	5	High
Tye, 2006 (29)	1	1	1	0	0	1	4	Medium
Urbaniok, 2007 (47)	1	0	1	1	1	0	4	Medium

First Author, Year (Reference No.)	Represen-tativeness	Sample size	Reporting of participation rate	Satisfactory rate of participation	Validity of diagnostic	Quality of statistics	Total score	Quality group
Vicens, 2011 (46)	1	1	1	1	1	1	6	High
von Schönfeld, 2006 (39)	1	0	1	0	1	1	4	Medium
Zabala-Baños, 2016 (45)	1	1	1	1	1	1	6	High
Zlotnick, 1997 (56)	1	0	1	0	0	1	3	Medium

Abbreviations: F, female; M, male; MQ, Martinique

Web Table 2. Data Extracted from the Included Samples, 1980-2017

First Author, Year (Reference, No.)	Final year of data collection	Sample size	Refusal Rate (%)[b]	Mean Age (years)	Number of PTSD Cases		
					Point	One-year	Lifetime
Andreoli, 2014 (57) M, sentenced	2007	676		32.2	37	53	178
Andreoli, 2014 (57) F, sentenced	2007	617	5.8	32.1	89	99	248
Andreoli, 2014 (57) M remand	2007	516		27.8	41	57	172
Assadi, 2006 (60)	2003	351	12.2	32.7	2		
Beaudette, 2016 (31)	2014	1110	22.0	35.6	122		149
Bebbington, 2017 (48) M	2009	197	22.9	34.6	9		
Bebbington, 2017 (48) F	2009	171	22.8	31.8	20		
Boşgelmez, 2010 (61) M	2005	30	6.3	30.1	2		
Boşgelmez, 2010 (61) F	2005	30	11.8	31.1	3		
Brink, 2001 (32)	1999	202	1.5	33.0	8		10
Brooke, 1996 (49)	1993[a]	750	18.0	27.5	13		
Bulten, 2009 (43)	2006[a]	191	18.7	30.4	9		
Butler, 2003 (28) M, mixed	2001	756		29.6	128	164	
Butler, 2003 (28) M, sentenced	2001	458	15.0	33.8	43	73	
Butler, 2003 (28) F, mixed	2001	165		29.1	62	72	
Butler, 2003 (28) F, sentenced	2001	108	16.0	32.7	30	46	
Derkzen, 2016 (34)	2016	154		36.0	51		53
Duburcq, 2004 (36) M	2004	799	39.4	39.0	66		
Duburcq, 2004 (36) M, MQ	2004	100	45.0	38.0	13		
Duburcq, 2004 (36) F	2004	99	50.0	38.5	13		
Dudeck, 2009 (37)	2007	102	15.0	31.2	3		6
Einarsson, 2009 (41)	2006[a]	90	5.7	31.0	4		
Gunter, 2008 (50) M	2005[a]	264	0.6	31.1	27		
Gunter, 2008 (50) F	2005[a]	56		31.1	13		

First Author, Year (Reference, No.)	Final year of data collection	Sample size	Refusal Rate (%)[b]	Mean Age (years)	Number of PTSD Cases		
					Point	One-year	Lifetime
Guthrie, 1998 (51)	1998	100	46.8	37.9	14		30
Hodgins, 1990 (33)	1988	495	21.2	31.0			64
Huang, 2006 (59)	2004	471	0.0	31.6	50		75
Lynch, 2014 (52) sentenced	2012	233	22.4	35.0		73	136
Lynch, 2014 (52) remand	2012	249				66	120
Math, 2011 (21) sentenced	2009	1197		38.0	1		
Math, 2011 (21) remand	2009	3827		28.4	13		
Mir, 2015 (40)	2013	150	24.2	34.3	39		
Missoni, 2003 (38)	2000	107	2.9	34.5	4		
Mohan, 1997 (42)	1993[a]	45	0.0	25.8	1		
Mundt, 2013 (58) M	2007	855	1.00	32.3		6	
Mundt, 2013 (58) F	2007	153		34.2		5	
Mundt, 2016 (35) M	2013	229	7.0	30.0	43		
Mundt, 2016 (35) F	2013	198		33.5	32		
Naidoo, 2012 (20) sentenced	2009	120		30.5	6		
Naidoo, 2012 (20) remand	2009	73		30.5	13		
Powell, 1997 (53) sentenced	1994[a]	118	29.7	32[c]	32		69
Powell, 1997 (53) remand	1994[a]	95			13		
Simpson, 1999 (44) M, sentenced	1998	645	22.0	30.6	55		124
Simpson, 1999 (44) M, remand	1998	441	18.3	28.5	42		100
Simpson, 1999 (44) F, sentenced	1998	162	19.0	29.3	27		60
Stompe, 2010 (30) sentenced	2007[a]	100			2		
Stompe, 2010 (30) remand	2007[a]	100			2		
Teplin, 1996 (54)	2003	1272	4.2	28.0	284		426
Trestman, 2007 (55) M	2004[a]	306	7.0	31.6	17		61
Trestman, 2007 (55) F	2004[a]	199			43		83
Tye, 2006 (29)	2000	103	22.0	29.6		33	

First Author, Year (Reference, No.)	Final year of data collection	Sample size	Refusal Rate (%)[b]	Mean Age (years)	Number of PTSD Cases		
					Point	One-year	Lifetime
Urbaniok, 2007 (47)	2004	25	10.7		8[d]		
Vicens, 2011 (46)	2008	707	9.7	36.8	3		25
von Schönfeld, 2006 (39)	2003	63	29.5	33.9	20		29
Zabala-Baños, 2016 (45)	2012	184	2.3	39.6	11		42
Zlotnick, 1997 (56)	1994[a]	85	25.0	31.0	41		58

Abbreviations: F, female; M, male; MQ, Martinique; PTSD, post-traumatic stress disorder.

[a] Year of data collection is imputed based on average mean difference between publication year and year of data collection of the other samples.

[b] When refusal rate was not available, non-response rates were used.

[c] Median.

[d] Conservative estimation.

Web Table 3. Sensitivity Analysis

	Gender	Quality scoring	Prevalence Rate, % (95% CI)		I-squared, % (95% CI)
Point prevalence of PTSD	Male	All	6.2	(95% CI: 3.9, 9.0)	97% (95% CI: 97, 98)
		High quality	5.9	(95% CI: 4.0, 8.2)	92% (95% CI: 90, 94)
	Female	All	21.1	(95% CI: 16.9, 25.6)	90% (95% CI: 85, 94)
		High quality	18.0	(95% CI: 14.2, 22.1)	87% (95% CI: 77, 92)
One-year prevalence of PTSD	Male	All	9.9	(95% CI: 3.0, 20.2)	99% (95% CI: 98, 99)
		High quality	7.6	(95% CI: 1.8, 16.9)	98% (95% CI: 97, 99)
	Female	All	26.1	(95% CI: 15.9, 37.8)	96% (95% CI: 93, 97)
		High quality	17.7	(95% CI: 3.8, 38.6)	97% (95% CI: 94, 99)
Lifetime prevalence of PTSD	Male	All	17.8	(95% CI: 12.4, 23.9)	97% (95% CI: 96, 98)
		High quality	17.3	(95% CI: 9.5, 26.8)	98% (95% CI: 97, 98)
	Female	All	40.4	(95% CI: 31.8, 49.3)	96% (95% CI: 94, 97)
		High quality	33.1	(95% CI: 24.0, 42.8)	96% (95% CI: 93, 98)

Abbreviations: CI, confidence interval; PTSD, post-traumatic stress disorder.

Web Figure 1. Funnel Plots of Point Prevalence Estimates Against Standard Errors (for male and female samples), 1980-2017

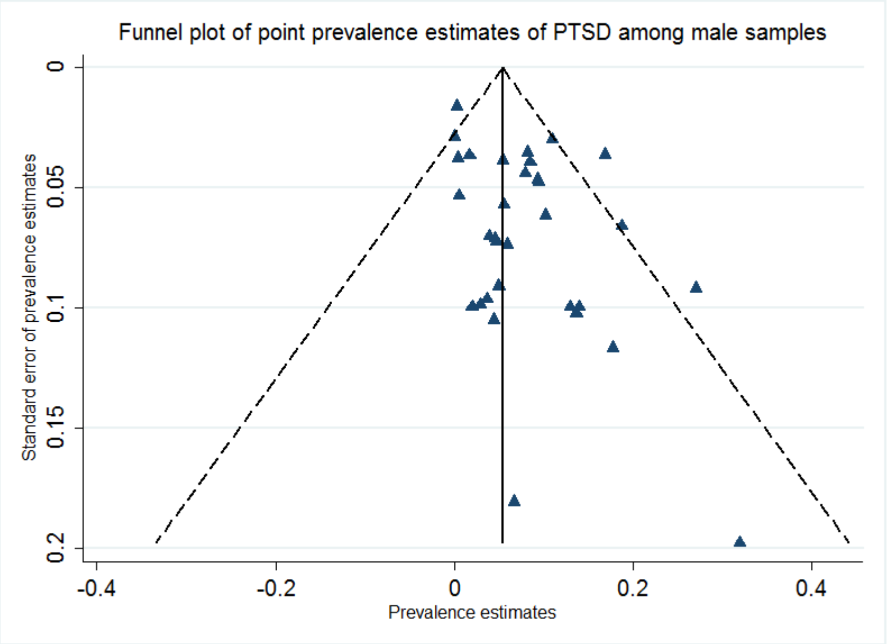

Legend: Egger’s test suggested a significant bias in the male samples reporting point prevalence of PTSD (*bias* = 1.047, SE(*bias*) = 0.376; (*P* = 0.009). PTSD, post-traumatic stress disorder.

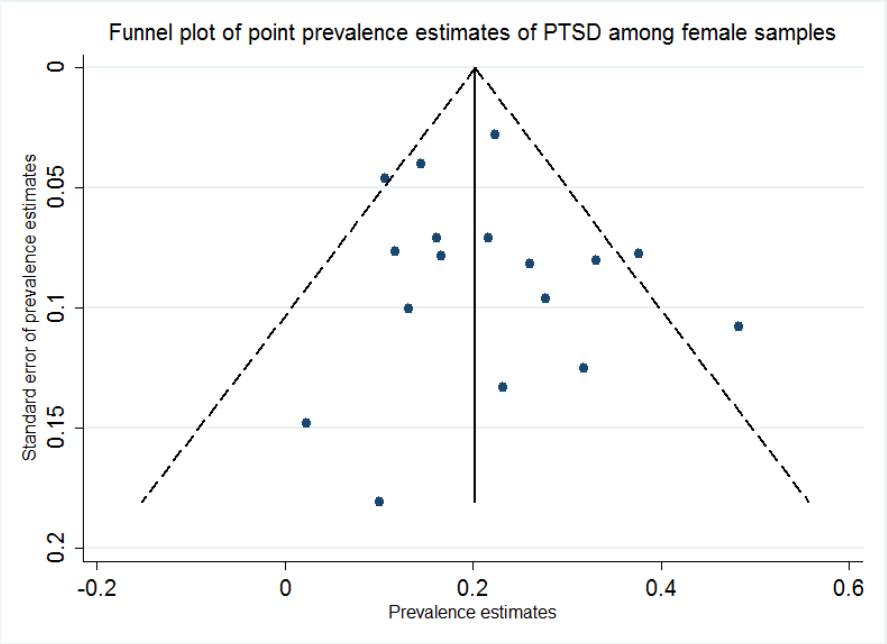

Legend: Egger’s test suggested no significant bias in the female samples reporting point prevalence of PTSD (*bias* = 0.486, SE(*bias*) = 0.681; *P* = 0.49). PTSD, post-traumatic stress disorder.

Web Figure 2. Funnel Plots of One-year Prevalence Estimates Against Standard Errors (for male and female samples), 1980-2017

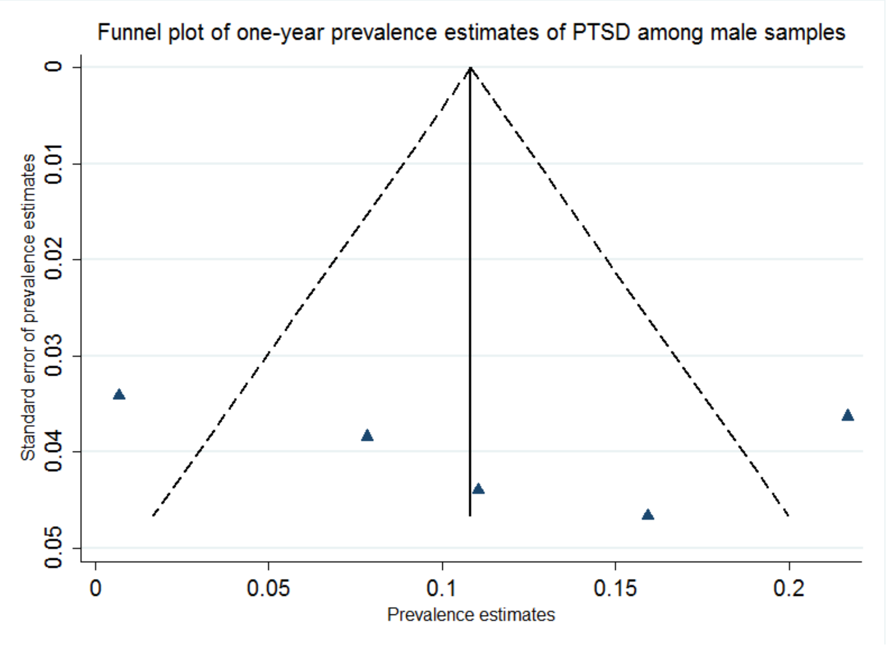

Legend: Egger's test suggested no significant bias in the male samples reporting one-year prevalence of PTSD (*bias* = 6.072, SE(*bias*) = 9.338; *P* = 0.56). PTSD, post-traumatic stress disorder.

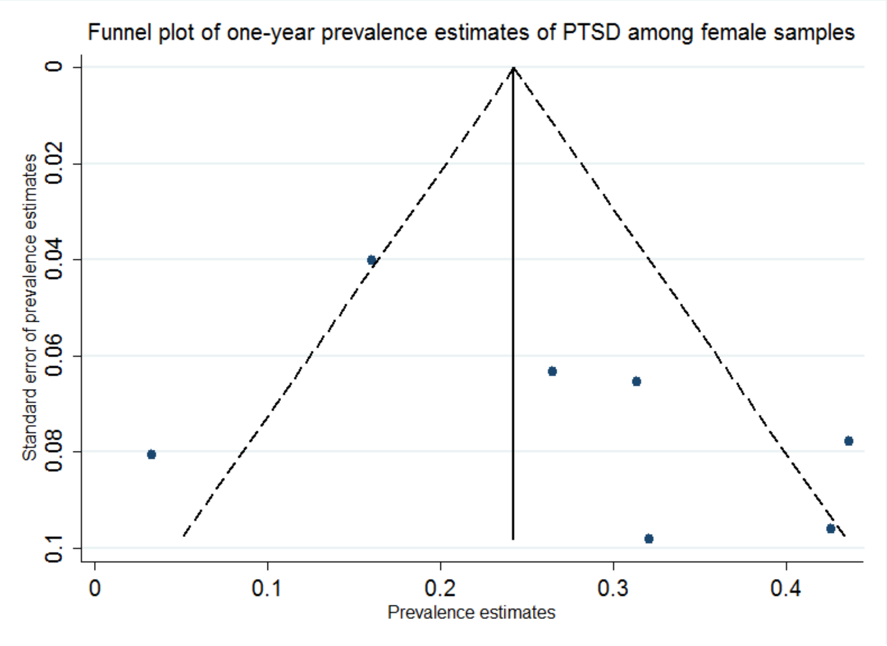

Legend: Egger's test suggested no significant bias in the female samples reporting one-year prevalence of PTSD (*bias* = 3.127, SE(*bias*) = 2.240; *P* = 0.22). PTSD, post-traumatic stress disorder.

Web Figure 3. Funnel Plots of Lifetime Prevalence Estimates Against Standard Errors (for male and female samples), 1980-2017

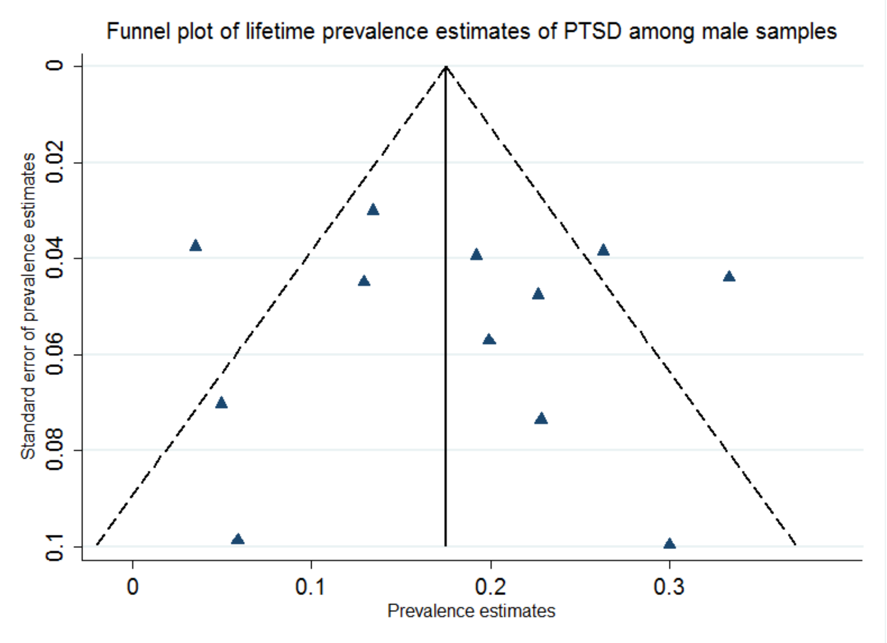

Legend: Egger's test suggested no significant bias in the male samples reporting lifetime prevalence of PTSD (*bias* = 0.565, SE(*bias*) = 1.840; *P* = 0.78). PTSD, post-traumatic stress disorder.

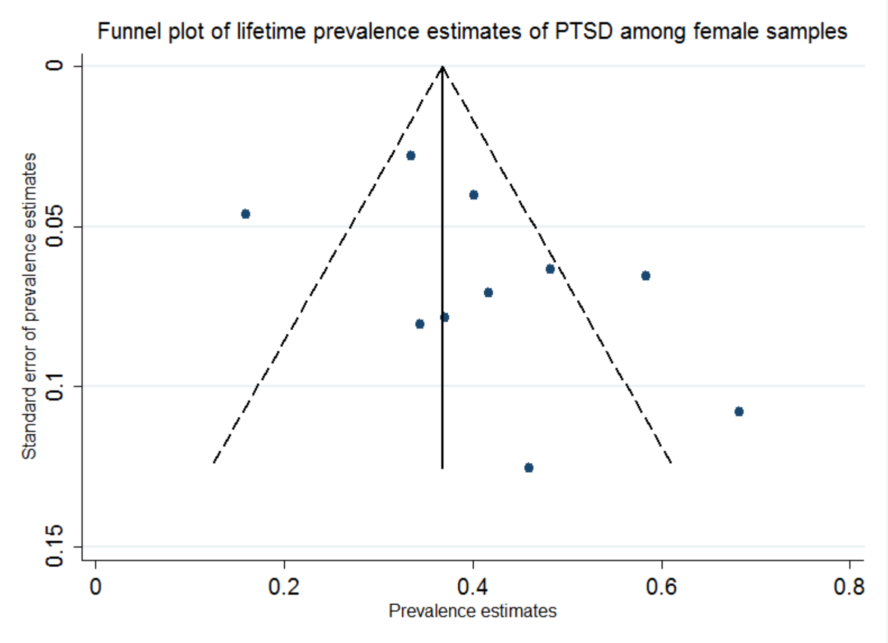

Legend: Egger's test suggested no significant bias in the female samples reporting lifetime prevalence of PTSD (*bias* = 2.451, SE(*bias*) = 1.580; *P* = 0.16). PTSD, post-traumatic stress disorder.
